# Supplementary material for: External Validation of Risk Scores for Predicting Venous Thromboembolism in Ambulatory Patients with Lung Cancer
Source: Cancers (Basel). 2024 Sep 15;16(18):3165. doi: 10.3390/cancers16183165 (PMC11430721; doi:10.3390/cancers16183165)
Supplement: Supplementary file 1 [file cancers-16-03165-s001.zip › cancers-3127506-supplementary.pdf]

Supplementary Table S1 Models for predicting VTE in ambulatory patients with lung cancer

Name of model (first author, year)	Cancer types for model derivation	Predictors	Score	High risk
Khorana Score [1]	various	Cancer tissue:<br>• Very high-risk site (stomach, pancreas)<br>• High risk site (lung, lymphoma, gynaecologic, bladder, testicular)<br>Platelet count ≥ 350 X 10^9/L<br>Haemoglobin < 10 g/dL and/or use of ESA<br>Leukocyte count >11 X 10^9/L<br>BMI ≥ 35 kg/m^2	 2<br>1<br><br>1<br>1<br>1<br>1	Score ≥ 3 [#]
PROTECHT Score [2]	various	As Khorana Score, but<br>**add** Gemcitabine chemotherapy, or<br>Platinum chemotherapy	 1	Score ≥ 3
CONKO Score [3]	various	As Khorana Score, but<br>**remove** BMI ≥ 35 kg/m^2, and<br>**add** ECOG PS ≥ 2	 <br>1	Score ≥ 3
COMPASS-CAT Score [4]	Various<br>(13% lung cancer)	Anti-hormonal therapy or anthracycline treatment<br>Time since cancer diagnosis ≤ 6 months<br>Central venous catheter<br>Advanced stage of cancer<br>Cardiovascular risk factors present<br>Recent hospitalization for acute medical illness<br>A history of VTE<br>Platelets count ≥ 350 X 10^9/L	6<br>4<br>3<br>2<br>5<br>5<br>1<br>2	Score ≥ 7 [†]

ESA: erythropoiesis stimulating agents; BMI: body mass index; ECOG PS: Eastern Cooperative Oncology Group Performance Status.

[#] Score ≥ 2 was used to stratify high risk patients in the CASSINI clinical trial of primary thromboprophylaxis [5] and has been incorporated into ASCO Guideline [6].

[†] Score ≥ 11 was proposed in the study by Rupa-Matysek, J., et al. [7]

References

1. Khorana, A.A.; Kuderer, N.M.; Culakova, E.; Lyman, G.H.; Francis, C.W. Development and validation of a predictive model for chemotherapy-associated thrombosis. *Blood* **2008**, *111*, 4902–4907.
2. Verso, M.; Agnelli, G.; Barni, S.; Gasparini, G.; LaBianca, R. A modified Khorana risk assessment score for venous thromboembolism in cancer patients receiving chem-otherapy: The Protecht score. *Intern. Emerg. Med.* **2012**, *7*, 291–292.
3. Pelzer, U.; Sinn, M.; Stieler, J.; Riess, H. Primary pharmacological prevention of thromboembolic events in ambulatory patients with advanced pan-creatic cancer treated with chemotherapy? *Dtsch. Med. Wochenschr.* **2013**, *138*, 2084–2088.
4. Gerotziafas, G.T.; Taher, A.; Abdel-Razeq, H.; AboElnazar, E.; Spyropoulos, A.C.; El Shemmari, S.; Larsen, A.K.; Elalamy, I.; on behalf of the COMPASS–CAT Working Group. A Predictive Score for Thrombosis Associated with Breast, Colorectal, Lung, or Ovarian Cancer: The Prospective COMPASS–Cancer-Associated Thrombosis Study. *Oncologist* **2017**, *22*, 1222–1231.
5. Khorana, A.A.; Soff, G.A.; Kakkar, A.K.; Vadhan-Raj, S.; Riess, H.; Wun, T.; Streiff, M.B.; Garcia, D.A.; Liebman, H.A.; Belani, C.P.; et al. Rivaroxaban for Thromboprophylaxis in High-Risk Ambulatory Patients with Cancer. *N. Engl. J. Med.* **2019**, *380*, 720–728.
6. Key, N.S.; Khorana, A.A.; Kuderer, N.M.; Bohlke, K.; Lee, A.Y.; Arcelus, J.I.; Wong, S.L.; Balaban, E.P.; Flowers, C.R.; Gates, L.E.; et al. Venous Thromboembolism Prophylaxis and Treatment in Patients With Cancer: ASCO Guideline Update. *J. Clin. Oncol.* **2023**, *41*, 3063–3071.
7. Rupa-Matysek, J.; Lembicz, M.; Rogowska, E.K.; Gil, L.; Komarnicki, M.; Batura-Gabryel, H. Evaluation of risk factors and assessment models for predicting venous thromboembolism in lung cancer patients. *Med. Oncol.* **2018**, *35*, 63.
